# Association of serum PUFA and linear growth over 12 months among 6–10 years old Ugandan children with or without HIV

**DOI:** 10.1017/S1368980022000611

**Published:** 2022-05

**Authors:** Ruth A Pobee, Jenifer I Fenton, Alla Sikorskii, Sarah K Zalwango, Isabella Felzer-Kim, Ilce M Medina, Bruno Giordani, Amara E Ezeamama

**Affiliations:** 1 Department of Food Science and Human Nutrition, Michigan State University, East Lansing, MI, USA; 2 Department of Psychiatry, Michigan State University, 909 Wilson Road, 322B West Fee Hall, East Lansing, MI 48824, USA; 3 Directorate of Public Health and Environment, Kampala Capital City Authority, Kampala, Uganda; 4 College of Human Medicine, Michigan State University, East Lansing, MI, USA; 5 Department of Biosystems and Agricultural Engineering, Michigan State University, East Lansing, MI, USA; 6 Departments of Psychiatry, Neurology and Psychology, University of Michigan, Ann Arbor, MI, USA

**Keywords:** Perinatal HIV exposure, PUFA, Linear growth, Children

## Abstract

**Objective:**

To quantify PUFA-associated improvement in linear growth among children aged 6–10 years.

**Design:**

Serum fatty acids (FA), including essential FA (EFA) (linoleic acid (LA) and *α*-linolenic acid (ALA)) were quantified at baseline using GC-MS technology. FA totals by class (*n*-3, n-6, *n*-9, PUFA and SFA) and FA ratios were calculated. Height-for-age *Z*-score (HAZ) relative to WHO population reference values were calculated longitudinally at baseline, 6 and 12 months. Linear regression models estimated PUFA, HIV status and their interaction-associated standardised mean difference (SMD) and 95 % CI in HAZ over 12 months.

**Setting:**

Community controls and children connected to community health centre in Kampala, Uganda, were enrolled.

**Participants:**

Children perinatally HIV-infected (CPHIV, *n* 82), or HIV-exposed but uninfected (CHEU, *n* 76) and community controls (*n* 78).

**Results:**

Relative to highest FA levels, low SFA (SMD = 0·31, 95 % CI: 0·03, 0·60), low Mead acid (SMD = 0·38, 95 % CI: 0·02, 0·74), low total *n*-9 (SMD = 0·44, 95 % CI: 0·08, 0·80) and low triene-to-tetraene ratio (SMD = 0·42, 95 % CI: 0·07, 0·77) predicted superior growth over 12 months. Conversely, low LA (SMD = -0·47, 95 % CI: −0·82, −0·12) and low total PUFA (sum of total *n*-3, total *n*-6 and Mead acid) (SMD = -0·33 to −0·39, 95 % CI: −0·71, −0·01) predicted growth deficit over 12 months follow-up, regardless of HIV status.

**Conclusion:**

Low *n*-3 FA (ALA, EPA and *n*-3 index) predicted growth deficits among community controls. EFA sufficiency may improve stature in school-aged children regardless of HIV status. Evaluating efficacy of diets low in total SFA, sufficient in EFA and enriched in *n*-3 FA for improving child growth is warranted.

HIV infection and malnutrition are significant public health problems that affect 1·6 and 340 million children from Africa, respectively^(1)^. Children with perinatally acquired HIV infection (CPHIV) are especially vulnerable to stunting due to HIV-specific morbidity and a high prevalence of nutritional deficiencies^(2,3)^. Malnourished CPHIV are at elevated risk of death relative to HIV-uninfected peers^(4)^. Access to antiretroviral therapy (ART) contributes to improved growth among CPHIV^(5)^, but immune restoration is seldom complete^(6)^ and growth disadvantages persist in early childhood^(5)^. Through 24 months of life, even children exposed to HIV but uninfected (CHEU) have worse growth outcomes compared to children who are HIV unexposed and uninfected (CHUU, control children)^(7)^. Monitoring of long-term growth in HIV-affected children remains important to understand growth trajectory and to identify modifiable intervention.

Stunting, a form of malnutrition that represents poor growth secondary to chronic protein–energy malnutrition (PEM), affects an estimated 144 million African children^(8,9)^. PEM is conceptually defined as an imbalance between the supply of dietary protein and total energetic intake^(10)^. However, PEM does not consider the source of dietary lipids nor the fatty acid (FA) composition of dietary lipids. This is a limitation given that essential fatty acids (EFA) are those FA that can only be derived from dietary sources. The two EFA in humans include *α*-linolenic acid (ALA; an *n*-3 FA) and linoleic acid (LA; an *n*-6 FA). They are important for storage and transport of energy, cell signalling, bone development, brain myelination, and hormone synthesis and are substrates for signalling molecules^(11–14)^. ALA and LA are substrates for endogenous synthesis of long-chain PUFA such as EPA, DHA and arachidonic acid (AA) that have important roles in cell differentiation, cell growth, cell membrane structure, gene expression and signal transduction^(11–13)^. Hence, essential fatty acid deficiency (EFAD) has been implicated in the pathophysiology of stunting, although robust epidemiologic data are limited^(15)^.

The importance of EFA sufficiency for early child growth and neurodevelopment is well established^(16)^, but its role in long-term child growth is less clear. In cross-sectional studies of children between 2 and 6 years old from Ghana^(17)^ and Tanzania^(18)^, serum EFA and total *n*-6 FA were positively associated with growth, serum Mead acid, a FA synthesised when EFA are low, was inversely associated with growth and *n*-3 FA class were not associated with growth. Similarly, an inverse association was reported between growth and triene-to-tetraene (T/T) ratio (a metric indicative of EFAD) among children from Tanzania^(18)^, although this association was not confirmed in Ghanaian children^(17,19)^. Among Ugandan children with and without HIV infection or exposure^(20)^, we have reported inverse associations between T/T ratio and several growth parameters (HAZ, weight-for-age and BMI-for-age), but the prospective relationship between FA and child growth has not been specifically examined. The present study addresses this knowledge gap. Specifically, we assess the associations between several FA and growth over 12 months in CPHIV, CHEU and CHUU and evaluate perinatal HIV status as a mediator and potential moderator. We hypothesised that high EFA (ALA and LA), high *n*-3 FA and low SFA would be associated with superior growth. We further hypothesised that PHIV children would show inferior growth compared to CHEU and CHUU children, and that this association would be independent of FA level. Finally, we hypothesised that the magnitude and/or direction of association between respective FA and growth may differ according to EFA and non-EFA classes within perinatal HIV groups.

## Methods

### Study sample

Three hundred and five Ugandan children (6–10 years old) with and without HIV infection were enrolled from the Kawaala Health Center (KHC) in Kawempe Division of Kampala, Uganda, as part of a 12-month prospective cohort study^(20)^. Of these, serum levels of PUFA were available for 237 children including perinatally acquired HIV infection (CPHIV, *n* 82), perinatally HIV exposed but uninfected (CHEU, *n* 76) and HIV unexposed uninfected (CHUU, *n* 78) community control children that constituted the study sample for this investigation.

### Exclusion/inclusion criteria

Eligibility criteria for the parent study which also applied to this nested study included as follows: being between the ages of 6 and 10 years at enrolment, documented record of being born in a hospital/healthcare setting, and availability of medical record data to objectively establish HIV status during pregnancy and time of child’s birth for index child and their birth mother. Children born in non-clinic settings, children lacking official birth records or who were missing antenatal register/delivery medical records were excluded, because objective HIV status could not be established for birth mother and enrolled child. Additional eligibility criteria for this secondary analysis included availability of FA and growth data. HIV-negative status was confirmed for CHEU and CHUU at enrolment via HIV rapid diagnostic test.

### Measures

#### Growth measures

Height was measured in centimetres using a stadiometer, while weight was measured in kilograms using a calibrated beam balance scale (Seca Classic Beam Scale, Model#700, Seca Inc.) at baseline, 6 and 12 months. At each time point, both height and weight measures were taken in triplicate and the mean values were recorded. Three growth indicators (weight-for-height, BMI-for-age and height-for-age) were used and metrics: weight-for-age *Z*-score (WAZ), BMI for-age *Z*-score (BAZ) and height-for-age *Z*-score (HAZ) were calculated using WHO AnthroPlus macro^(21)^. The growth metrics to measure growth were changes in WAZ, BAZ and HAZ. Long-term stature, defined per HAZ, was evaluated as the continuous outcome covariate of primary interest.

#### Serum fatty acid

Serum FA was measured at baseline only using venous blood. Whole blood was centrifuged to obtain serum. Serum was stored at −80^°^C until shipped on dry ice to the US laboratory for FA analyses. Of the total samples shipped, 237 were analysable for FA. FA were extracted and methylated from serum as previously described^(20)^. A modified methylation described by Jenkins (2010) was conducted^(22)^. Briefly, a 100-μl aliquot of serum with 20 µg of internal standard (methyl 12-tridecenoate, U-35M, Nu-Chek Prep) suspended in isooctane. Two millilitres of 0·5 N anhydrous potassium methoxide was added and samples were heated at 50°C for 10 min. Once cool, 3 ml of 5 % methanolic HCl was added, and samples were heated at 80°C for 10 min. Once cool, 2 ml of water and 2 ml of hexane were added, and the upper organic phase was removed and dried to obtain fatty acid methyl esthers (FAME). A PerkinElmer 680/600S GC-MS in the electron impact mode (70 eV) was equipped with an Agilent Technologies HP-88 column (100 m, 0·25 mm ID and 0·2 µM film thickness) for FAME quantification as described by Kramer *et al*. 2008^(23)^. The MS data were recorded in full scan mode (mass range of m/z 70–400 amu). MS transfer line and ion source temperature were set at 180°C. Quantification of FAME was conducted using internal standard and chromatographic peak area, and FA were reported as percent of total FA quantified as previously described^(20)^. A GC reference standard was created by combining Supelco 37 Component FAME Mix (Sigma-Aldrich) with Mead acid, docosatetraenoic acid, *n*-3 docosapentaenoic acid (DPA), *n*-6 DPA and palmitelaidic acid purchased from Cayman Chemical (Ann Arbor, MI). Data analysis was conducted using MassLynx V4.1 SCN 714 (Waters Corporation). FA totals by class (*n*-3, *n*-6, *n*-9, PUFA and SFA), and FA ratios (*n*-6/*n*-3 ratio and T/T ratio) at baseline were calculated as defined. In the absence of clinically defined threshold for FA deficiency, four equal categories based on sample quartiles were initially defined for each FA. Further collapsing of quartiles to two or three levels was informed by the magnitude/direction of crude relationship between respective FA and HAZ. For example, the magnitude and direction of unadjusted association with HAZ was similar for quartile 1 *v*. 4 and quartile 2 *v*. 4 for LA. Therefore, LA was analysed in three categories, that is, combined quartiles 1 and 2, quartile 3, and quartile 4. For each FA, difference in growth outcomes were calculated for one to three lower categories relative to the highest category.

### Other covariates

#### Perinatal HIV status

Child perinatal HIV status was defined on the basis of HIV mother-to-child transmission as children perinatally HIV infected (CPHIV), HIV-exposed uninfected (CHEU) and children HIV unexposed uninfected (CHUU). CPHIV and CHEU status by breast-feeding cessation were, respectively, verified using a positive and negative DNA-PCR tests, respectively. Current HIV-negative status at study enrolment was confirmed for CHEU and CHUU via a negative HIV-rapid diagnostic test.

#### Hb

Hb was measured in grams per decilitre as part of complete blood cell count.

#### Caregiver sociodemographic characteristics

Age (in years), sex (male *v*. female), years of education, functioning in caregiving role per the Barkin index of maternal functioning^(24,25)^ and acute psychosocial stress per the perceived stress scale^(26)^ were defined at enrolment and adjusted as potential confounders in multivariable analyses, since maternal stress can affect childhood growth and development^(27)^.

### Statistical analyses

Differences in means per ANOVA for continuous measures and differences in proportion per chi-square tests, for categorical factors, was used to describe study population across HIV groups at baseline with respect to child/caregiver sociodemographic and child growth measures. Multivariable linear mixed effects models were implemented to quantify FA-related standardised mean differences (SMD) in age- and sex-standardised measures of growth over 12-month follow-up using SAS PROC MIXED. In all models, confounders such as caregivers’ age, sex, socio-economic status and psychosocial stress were adjusted based on subject matter knowledge. Random effect of the caregiver was included in all models to account for nesting of children within households. Time was entered as a class variable to model potentially non-linear patterns, and interaction between time and FA levels was examined to assess potential variation in rate of growth over 12 months according to FA levels. The least square-adjusted means according to FA levels were output from the linear mixed effects (LME) models, and differences among them were tested at each time point. When there was no appreciable time trend, the least square means were averaged over time within the LME model, and differences in growth according to FA categories were tested. Since growth measures are standardised by age and sex, SMD are estimated – a measure comparable to Cohen’s effect size and thus permitting determination of clinical importance. Per prior precedent based on studies of quality of life, SMD of ≥|0·33| were deemed clinically important for child growth which has wide-ranging lifelong implications^(28)^. Hence, SMD<|0·33|, |0·33|≤SMD<|0·50| and SMD≥|0·50| are, respectively, considered to be of small to modest, moderate and large clinical importance in this study.

To evaluate perinatal HIV status as a potential mediator of FA-related differences in growth, multivariable regression models already adjusted for demographic and psychosocial factors were sequentially adjusted for perinatal HIV status. Lastly, in addition to testing for main effect of FA and evaluating perinatal HIV status as a potential mediator, the potential for heterogeneity in FA relationship to growth by HIV (HIV × FA) was evaluated. In the absence of heterogeneity, results are presented for the overall sample. Potential heterogeneity was indicated when *P*-value for HIV × FA was <0·1. In that case, results for FA-related SMD in growth are presented separately according to perinatal HIV status categories. All analyses were performed with SAS version 9.4 (SAS Institute, Inc.). All hypotheses tests were two-sided at *α* = 0·05.

## Results

### Descriptive analyses

The average age of children was 7·69 (sd 1·44) years and included 46 % (*n* 109) girls. Average age and proportion of girls to boys were similar by HIV status. Overall, growth measures were lower (−0·55 to −0·73) for Ugandan children relative to WHO age/sex reference growth values. Baseline HAZ differed according to perinatal HIV status with CPHIV having significantly lower HAZ and WAZ values than both CHEU and CHUU. There was no difference in HAZ for CHEU compared to CHUU. Mean Hb was 13·07 (sd 1·31) g/dl and did not differ by HIV status. Approximately 41 % of the caregivers had no or less than elementary school education. Years of education was greater for caregivers of CHUU relative to caregivers of HIV-affected children (Table 1).Growth values at months 6 and 12 were, respectively, available for 93 % and 97 % of the 237 children included at baseline in this longitudinal study (data not shown).

**Table 1:** Sociodemographics and clinical characteristics of Ugandan children 6–10 years by HIV status

Demographics	Overall			CPHIV			CHEU			CHUU			*P*-value
	*n*	Mean	sd	*n*	Mean	sd	*n*	Mean	sd	*n*	Mean	sd	
Age (years)	236	7·69	1·44	82	7·82	1·55	76	7·54	1·35	78	7·72	1·44	0·48
		%			%			%			%		
Sex													
Female	109	46·19		39	47·56		38	50·00		32	41·03		0·51
Male	127	53·81		43	52·44		38	50·00		46	58·97		
Height (cm)	237	124·13	9·78	82	122·58	9·57	76	124·45	10·04	78	125·45	9·69	0·17
Weight (kg)	237	23·51	5·11	82	23·09	5·15	76	23·41	4·80	78	24·03	5·39	0·50
HAZ	237	−0·55	1·08	82	−0·92	0·89	76	−0·35	1·16	78	−0·35	1·09	0·001
WAZ	205	−0·73	1·06	67	−0·98	0·99	69	−0·63	1·11	69	−0·60	1·06	0·07
BAZ	237	−0·62	1·05	82	−0·56	0·96	76	−0·64	1·05	78	−0·66	1·16	0·82
Hb (g/dl)	234	13·07	1·31	82	12·89	1·30	75	13·08	1·55	77	13·26	1·02	0·20
Serum albumin (g/dl)	236	45·61	2·78	82	46·25	2·90	76	44·99, 2·70		78	45·55, 2·63		0·017
	*n*	%		*n*	%		*n*	%		*n*	%		
Care giver education													
None/ < Elementary	94	40·87		32	40·51		37	50·00		25	32·47		0·039
Elementary completed	47	20·43		14	17·72		18	24·32		15	19·48		
Some/completed O’ levels	71	30·87		27	34·18		18	24·32		26	33·77		
Some A’ levels or more	18	7·83		6	7·59		1	1·35		11	14·29		

CPHIV, children with perinatally HIV infected; CHEU, children HIV exposed but uninfected; CHUU, HIV-unexposed and uninfected children; HAZ, height-for-age *Z*-score; WAZ, weight-for-age *Z*-score; BAZ, BMI-for-age *Z*-score.

Serum albumin as a surrogate measure of protein–energy malnutrition. ANOVA comparing mean fatty acid values across all HIV groups. Tukey’s *post hoc* test was used for mean difference; *X* ^2^ test was used to determine differences for categorical variables. Statistical significance at *P* < 0 05.

### Baseline fatty acids and growth over 12 months

SFA levels were inversely associated with growth after adjusting for time and caregiver sociodemographic factors. Specifically, the lowest *v*. highest baseline total SFA predicted superior growth of moderate clinical importance over 12 months (SMD = 0·38, 95 % CI: 0·09, 0·67). This association was affirmed for two individual SFA: behenic acid (SMD = 0·42, 95 % CI: 0·07, 0·76) and stearic acid (SMD = 0·49, 95 % CI: 0·21, 0·77). Further adjustment for perinatal HIV status weakened respective relationships, although the inverse association of Behenic acid (SMD = 0·34, 95 % CI: 0·00, 0·68) and total SFA (SMD = 0·31, 95 % CI: 0·03, 0·60) to growth over 12 months remained of modest to moderate clinical importance.

With respect to *n*-6 FA class, baseline lowest *v*. the highest LA (SMD = -0·60, 95 % CI: −0·95, −0·26) and total *n*-6 FA (SMD = -0·37, 95 % CI: −0·61, −0·13) were, respectively, associated with growth deficits over 12 months. Adjustment for perinatal HIV status reduced magnitude of respective associations, but the adverse association of respective FA to growth remained of moderate to large clinical importance. Unadjusted for perinatal HIV status, lower *v*. highest levels of several individual *n*-6 FA were inversely associated with growth including ϒ-linolenic acid (GLA, SMD = 0·36 to 0·37, 95 % CI: 0·03, 0·70), dihommo-ϒ-linolenic acid (DGLA, SMD = 0·44, 95 % CI: 0·07, 0·81), AA (SMD = 0·39, 95 % CI: 0·02, 0·76) and docosatetraenoic acid (DTA, SMD = 0·43, 95 % CI: 0·10, 0·75). However, each of these associations were attenuated by adjustment for perinatal HIV status to levels corresponding to small clinical importance (Table 2).

**Table 2:** The relationship between FA groups and height-for-age with or without adjustment for HIV status

Class	Adjusted for	Association†	Height-for-age				
			Model 1‡		Model 2§		FA × HIV interaction
			Standardised mean diff	95 % CI	Standardised mean diff	95 % CI	
SFA	Stearic	Q1 *v*. Q3	0·49	0·21, 0·77*	0·26	−0·05, 0·57	0·69
		Q2 *v*. Q3	0·27	−0·00, 0·55	0·23	−0·04, 0·50	
	Behenic	Q1 *v*. Q4	0·42	0·07, 0·76*	0·34	0·00, 0·68*	0·14
		Q2 *v*. Q4	0·16	−0·20, 0·52	0·13	−0·23, 0·49	
		Q3 *v*. Q4	0·27	−0·06, 0·59	0·20	−0·13, 0·53	
		Q2 *v*. Q3	0·24	−0·08, 0·57	0·28	−0·04, 0·59	
*n*-3	DHA	Q1 *v*. Q2–4	−0·09	−0·38, 0·21	−0·14	−0·44, 0·15	0·22
*n*-6	Linoleic	Q1, 2 *v*. Q4	−0·60	−0·95, −0·26*	−0·47	−0·82, −0·12*	0·49
		Q3 *v*. Q4	−0·17	−0·50, 0·16	−0·11	−0·43, 0·22	
	GLA	Q1 *v*. Q4	0·21	−0·14, 0·55	0·09	−0·25, 0·43	0·43
		Q2 *v*. Q4	0·37	0·05, 0·70*	0·23	−0·08, 0·54	
		Q3 *v*. Q4	0·36	0·03, 0·68*	0·23	−0·09, 0·54	
	DGLA	Q1 *v*. Q4	0·44	0·07, 0·81*	0·08	−0·30, 0·46	0·45
		Q2 *v*. Q4	0·22	−0·10, 0·55	−0·06	−0·39, 0·26	
		Q3 *v*. Q4	0·18	−0·15, 0·52	−0·10	−0·43, 0·23	
	Arachidonic	Q1 *v*. Q4	0·07	−0·28, 0·41	−0·09	−0·45, 0·28	0·62
		Q2 *v*. Q4	0·39	0·02, 0·76*	0·26	−0·10, 0·62	
		Q3 *v*. Q4	0·17	−0·16, 0·51	0·13	−0·20, 0·46	
	DTA	Q1 *v*. Q4	0·43	0·10, 0·75*	0·25	−0·07, 0·57	0·82
		Q2 *v*. Q4	0·02	−0·30, 0·33	−0·11	−0·41, 0·20	
		Q3 *v*. Q4	0·13	−0·24, 0·50	−0·05	−0·41, 0·30	
Totals	Total *n*-3	Q1 *v*. Q4	0·02	−0·37, 0·41	−0·05	−0·42, 0·33	0·45
		Q2 *v*. Q4	0·04	−0·30, 0·37	0·04	−0·28, 0·36	
		Q3 *v*. Q4	0·01	−0·36, 0·38	0·02	−0·33, 0·37	
	Total *n*-6	Q1,2 *v*. Q3,4	−0·37	−0·61, −0·13*	−0·33	−0·56, −0·09*	0·79
	Total PUFA	Q1 *v*. Q4	−0·42	−0·79, −0·06*	−0·33	−0·65, −0·01*	0·71
		Q2 *v*. Q4	−0·39	−0·77, −0·02*	−0·39	−0·71, −0·08*	
		Q3 *v*. Q4	−0·06	−0·45, 0·34	−0·03	−0·40, 0·34	
	Total MUFA	Q1 *v*. Q4	0·18	−0·15, 0·51	0·17	−0·15, 0·50	0·63
		Q2 *v*. Q4	0·48	0·15, 0·81*	0·44	0·08, 0·80	
		Q3 *v*. Q4	0·04	−0·27, 0·35	0·02	−0·27, 0·31	
	Total SFA	Q1 *v*. Q3	0·38	0·09, 0·67*	0·31	0·03, 0·60*	0·98
		Q2 *v*. Q3	0·24	−0·08, 0·57	0·28	−0·04, 0·59	
*n*-9	Oleic	Q1 *v*. Q4	0·30	−0·04, 0·64	0·22	−0·11, 0·55	0·24
		Q2 *v*. Q4	0·38	0·01, 0·75*	0·34	−0·02, 0·69	
		Q3 *v*. Q4	0·27	−0·03, 0·58	0·21	−0·10, 0·51	
	Mead acid	Q1 *v*. Q4	0·54	0·18, 0·89*	0·38	0·02, 0·74*	0·68
		Q2 *v*. Q4	0·19	−0·13, 0·50	0·05	−0·28, 0·37	
		Q3 *v*. Q4	0·02	−0·32, 0·36	−0·03	−0·36, 0·31	
Ratios	T/T ratio	Q1 *v*. Q4	0·42	0·07, 0·77*	0·27	−0·08, 0·63	0·53
		Q2 *v*. Q4	0·21	−0·15, 0·57	0·09	−0·26, 0·21	
		Q3 *v*. Q4	−0·05	−0·40, 0·29	−0·13	−0·47, 0·21	
	*n*-6/*n*-3 ratio	Q1 *v*. Q4	−0·07	−0·44, 0·32	0·04	−0·33, 0·41	0·64
		Q2 *v*. Q4	−0·06	−0·45, 0·32	0·01	−0·37, 0·38	
		Q3 *v*. Q4	−0·01	−0·36, 0·34	0·05	−0·29, 0·38	

FA, fatty acid; FA × HIV, fatty acid by HIV interaction; GLA, ϒ-linolenic; DGLA, dihomo-*γ*-linolenic acid; DTA, docosatetraenoic; T/T ratio, triene-to-tetraene ratio.

Total SFA includes myristic, palmitic, arachidic, behenic and lignoceric acid; total *n*-3 includes *α* linolenic acid, EPA, docosapentaenoic acid and DHA; total PUFA is sum of total *n*-6, total *n*-3 and Mead acid; Q1: quartile 1 fatty acid; Q2: quartile 2 fatty acid; Q3: quartile 3 fatty acid; Q4: quartile 4 fatty acid (reference).

* Statistical significance at *P* < 0 05.

† All estimates are calculated from repeated measures linear mixed models.

‡ Model 1: Effect of FA on height-for-age adjusted for time and caregiver (age, education, caregiving quality and caregiver acute stress) but not HIV status.

§ Model 2: Effect of FA on height-for-age adjusted for HIV status plus for time and caregiver (age, education, caregiving quality and caregiver acute stress).

With respect to n-9 FA class, lower *v*. highest baseline oleic acid (SMD = 0·27 to 0·38, 95 % CI: −0·04, 0·75) and total MUFA (SMD = 0·03 to 0·44, 95 % CI: −0·27, 0·80) on average predicted higher growth over 12 months. However, neither of these associations were dose-dependent and after adjustment for perinatal HIV status, only inverse association between second *v*. fourth quartile of total MUFA persisted (SMD = 0·44, 95 % CI: 0·08, 0·80).

For Mead and FA class ratios in relationship to growth, lowest *v*. highest levels of Mead acid (SMD = 0·54, 95 % CI: 0·18, 0·89) and T/T ratio (SMD = 0·42, 95 % CI: 0·07, 0·77) were associated with moderate to clinically large growth advantage. However, only the association for Mead acid and growth was robust to adjustment for perinatal HIV status. The two lowest *v*. highest quartile of baseline total PUFA (SMD = -0·42 to −0·39, 95 % CI: −0·79, −0·02) on the other hand predicted growth deficit of moderate clinical importance over 12 months. This association remained clinically important though slightly attenuated (SMD = −0·39 to −0·33, 95 % CI: −0·71, −0·01) after adjustment for HIV status.

### Perinatal HIV status and growth over 12 months

Growth deficit of moderate to large clinical importance was evident for CPHIV in relationship to CHEU (SMD = −0·64, 95 % CI: −0·97, −0·32) or relative to CHUU (SMD = −0·48, 95 % CI: −0·76, −0·23), but growth trajectory was similar for CHEU (SMD = -0·04, 95 % CI:-0·24, 0·32) *v*. CHUU. These relationships were generally unaffected by sequential adjustment for individual FA. The relationship of perinatal HIV status to growth did not vary according to level of individual FA nor FA classes (HIV × FA interaction, *P*-value >0·10, Table 3).

**Table 3:** Effect of HIV on height-for-age with or without adjusting for FA

Class	Adjusted for	Association	Height-for-age		
			HIV related standardised mean differences in growth		HIV × FA interaction
			SMD	95 % CI*	
	Not adjusted for any FA†	CPHIV *v*. CHEU	−0·64	−0·97, −0·32	N/A
		CPHIV *v*. CHUU	−0·48	−0·76, −0·23	
Totals	Total SFA	CPHIV *v*. CHEU	−0·63	−0·74, −0·21	0·98
		CPHIV *v*. CHUU	−0·47	−0·95, −0·30	
	Total *n*-3	CPHIV *v*. CHEU	−0·65	−0·96, −0·33	0·45
		CPHIV *v*. CHUU	−0·49	−0·76, −0·21	
	Total *n*-6	CPHIV *v*. CHEU	−0·60	−0·91, −0·27	0·80
		CPHIV *v*. CHUU	−0·46	−0·70, −0·18	
	Total MUFA	CPHIV *v*. CHEU	−0·63	−0·94, −0·32	0·63
		CPHIV *v*. CHUU	−0·46	−0·72, −0·20	
	Total PUFA	CPHIV *v*. CHEU	−0·63	−0·95, −0·31	0·71
		CPHIV *v*. CHUU	−0·47	−0·74, −0·21	
*n*-9	Mead acid	CPHIV *v*. CHEU	−0·57	−0·89, −0·26*	0·22
		CPHIV *v*. CHUU	−0·43	−0·70, −0·15	
Ratio	T/T ratio	CPHIV *v*. CHEU	−0·59	−0·92, −0·27	0·53
		CPHIV *v*. CHUU	−0·44	−0·71, −0·18	

FA, fatty acid; SMD, standardised mean difference; FA × HIV, fatty acid by HIV interaction; CPHIV, children with perinatally HIV infected; CHEU, children HIV exposed but uninfected; CHUU, HIV-unexposed and uninfected children; T/T ratio, triene-to-tetraene ratio.

* Statistical significance at *P* < 0·05.

† Adjusting for time, child (age, sex) and caregiver (age, education, caregiving quality, caregiver acute stress).

Total *n*-3 includes *α*-linolenic acid, EPA, docosapentaenoic acid, *n*-3 and DHA; and total PUFA is sum of total *n*-6, total *n*-3 and Mead acid.

All estimates are calculated from repeated measures linear mixed models for effect of HIV on height-for-age adjusting for fatty acids plus for time, child age, child sex, caregiver (age, education, caregiving quality and caregiver acute stress.

### Relationship between some fatty acids and growth varied according to perinatal HIV status

The relationship between some FA and growth over 12 months varied according to perinatal HIV status (Table 4, FA × HIV, *P*-value <0·10). For example, among CPHIV only, lower *v*. highest level of arachidic acid (SMD = 0·54, 95 % CI: 0·03, 1·06) was associated with large growth advantage over 12 months. Likewise, the magnitude and direction of relationship between low nervonic acid and growth varied according to HIV status (*P*-value = 0·030), but relationships were not dose-dependent and this association was not statistically robust in any subgroup.

**Table 4:** Fatty acids related differences in height-for-age over 12 months within stratum of perinatal HIV status among 6–10 years old Ugandan children

			Perinatally HIV infected				Perinatally HIV exposed uninfected				HIV unexposed uninfected				
Fatty acids		Quartiles	Adjusted LSM	se	Mean diff	95 % CI	Adjusted LSM	se	Mean diff	95 % CI	Adjusted LSM	se	Mean diff	95 % CI	FA × HIV
SFA	Arachidic	Quartile 1	−0·60	0·24	0·54	0·03, 1·06*	0·01	0·21	0·47	−0·41, 1·34	−0·85	0·14	−0·41	−0·99, 0·16	0·027
		Quartile 2	−1·12	0·19	0·02	−0·43, 0·47	−0·43	0·23	0·02	−0·87, 0·90	−0·45	0·16	0·00	−0·59, 0·58	
		Quartile 3	−1·01	0·22	0·13	−0·36, 0·63	−0·74	0·22	−0·28	−1·15, 0·59	−0·35	0·21	0·10	−0·54, 0·74	
		Quartile 4	−1·15	0·12	Ref		−0·46	0·39	Ref		−0·45	0·26	Ref		
*n*-3	ALA	Quartile 1	−0·92	0·19	0·16	−0·29, 0·61	−0·02	0·27	0·33	−0·32, 0·97	−0·74	0·15	−0·38	−0·75, 0·00	0·10
		Quartile 2	−0·96	0·14	0·12	−0·29, 0·52	−0·74	0·15	−0·39	−0·94, 0·16	−0·69	0·24	−0·32	−0·85, 0·21	
		Quartiles (3, 4)	−1·08	0·15	Ref		−0·35	0·19	Ref		−0·37	0·13	Ref		
	EPA	Quartiles (1, 2)	−0·92	0·14	0·20	−0·23, 0·63	−0·41	0·19	−0·50	−1·08, 0·09	−0·62	0·13	−0·39	−0·88, 0·10	0·09
		Quartile3	−1·12	0·18	0·00	−0·49, 0·48	−0·91	0·22	−1·00	−1·63, −0·37*	−0·57	0·18	−0·34	−0·89, 0·20	
		Quartile 4	−1·12	0·18	Ref		0·09	0·24	Ref		−0·23	0·22	Ref		
	EPA + DHA	Quartile 1	−0·69	0·26	0·39	−0·16, 0·94	−0·41	0·26	−0·05	−0·63, 0·53	−0·77	0·14	−0·34	−0·69, 0·00	0·10
		Quartiles (2, 3, 4)	−1·08	0·11	Ref		−0·36	0·15	Ref		−0·43	0·12	Ref		
	*n*-3-index	Quartile 1	−0·74	0·25	0·35	−0·17, 0·87	−0·43	0·27	−0·08	−0·68, 0·52	−0·81	0·14	−0·37	−0·71, −0·02*	0·10
		Quartiles (2, 3, 4)	−1·09	0·10	Ref		−0·35	0·15	Ref		−0·44	0·13	Ref		
*n*-9	Nervonic	Quartile 1	−0·85	0·18	0·27	−0·16, 0·69	0·22	0·25	0·63	−0·02, 1·27	−0·63	0·19	−0·02	−0·45, 0·33	0·030
		Quartile 2	−1·16	0·20	−0·04	−0·46, 0·38	−0·75	0·15	−0·34	−0·82, 0·13	−0·27	0·22	0·34	−0·14, 0·84	
		Quartile 3	−0·89	0·15	Ref		−0·41	0·20	Ref		−0·61	0·13	Ref		

LSM, least square means; FA × HIV, fatty acid by HIV interaction; ALA, *α* linolenic acid.

* Statistical significance at *P* < 0·05.

FA × HIV <=0·1 was reported.

Neither DHA nor total *n*-3 FA levels were associated with growth over 12 months (Table 2). However, some FA in the *n*-3 class, that is, EPA, DHA, EPA + DHA and *n*-3 index, predicted growth in perinatal HIV-status-dependent manner. For example, low EPA levels predicted large growth deficits among CHEU, but this relationship was not dose-dependent with statistically robust association evident for CHEU in third *v*. fourth EPA quartile (SMD = −1·00, 95 % CI: −1·63, −0·37). Among CHUU, lower *v*. highest levels of these FA predicted growth deficits of moderate clinical importance over 12 months. The positive association between low *n*-3 FA and growth among CHUU was evident for ALA (SMD = -0·38, 95 % CI: −0·75, −0·00), EPA + DHA (SMD = -0·34, 95 % CI: −0·69, 0·00) and *n*-3 index (SMD = -0·37, 95 % CI: −0·71, −0·02).

## Discussion

### HIV-infected status exerts a sustained adverse impact on child growth

In this study of growth over 12 months among Ugandan children of 6–10 years old, CPHIV relative to CHEU or CHUU peers experienced significant growth disadvantage of moderate to large clinical importance. This hypothesis-consistent observation confirms prior observations of growth disadvantage for CPHIV^(29,30)^ relative to HIV-uninfected peers. We also provide novel prospective data relating variations in several FA classes with linear growth beyond 5 years of life among HIV-affected and unaffected children. Overlapping mechanisms such as antiretroviral therapy (ART) induced dysregulation of FA metabolism/profile^(31,32)^ and persistent gut permeability issues may act in concert to impede long-term linear growth among CPHIV^(33,34)^. The finding of overall similar growth trajectory over 12-month follow-up for CHEU and CHUU is reassuring, although possible differences in long-term growth trajectory for CHUU *v*. CHEU by peripartum ART exposure warrant further investigation.

### High levels of SFA is adversely associated with growth

Regardless of perinatal HIV status, low SFA (specifically, total SFA, palmitic and stearic) at baseline predicted superior growth of moderate clinical importance over 12 months. Furthermore, low arachidic acid was associated with moderate to large growth advantage over 12 months among HIV-affected children, although statistically significance was attained among CPHIV only. Support for these associations comes from widely recognised salutary effect of low SFA on cardiometabolic health parameters in adults^(35)^. Intervention studies comparing low total SFA diet to usual diet in children and adolescents reported beneficial impact on lipid profile without corresponding adverse impact on growth indices^(36)^. Findings from this longitudinal study confirm previously reported cross-sectional inverse association between total SFA and HAZ^(20)^ and suggest that diets low in total SFA will positively influence growth trajectory among children. Although SFA was inversely associated with growth in all children, more individual SFA, namely arachidic acid, are associated with poor growth among CPHIV. This observation is consistent with ART-induced dysregulation of FA metabolism among CPHIV or CHEU perinatally exposed to ART during sensitive developmental windows^(33,34)^. At least one study has reported FA dysregulation in ART-treated CHEU neonates, as evidenced by abnormal acylcarnitine profiles^(37)^. These dysregulations due to peripartum ART may have long-term growth consequences^(36–38)^, but specific studies are warranted to inform this knowledge gap in HIV-exposed children.

### Baseline essential fatty acid deficiency predicts worse growth

Serum measures indicative of EFAD include low ALA, low LA and elevated Mead acid and T/T ratio which is calculated as ratio of Mead acid to AA in the blood^(39)^. Mead acid is synthesised from oleic acid but only in the context of EFAD^(40)^. Five observations support of our study hypothesis that sufficiency in EFA will be associated with superior growth. First, children with lowest *v*. highest LA experienced moderate growth disadvantage over 12 months. Second, children in the lowest *v*. highest levels of Mead acid at baseline experienced moderate growth advantage over 12 months. Third, children in the two lowest *v*. highest quartile of baseline PUFA, a composite of three FA classes that includes Mead acid and LA, experienced moderate growth deficit over 12 months. Fourth, low T/T ratio was associated with moderate growth advantage over 12 months, although this association weakened significantly with adjustment for perinatal HIV status. Lastly, among CHUU only, low *v*. high baseline ALA predicted moderate growth deficit during follow-up. Given that EFA must be obtained from the diet to perform critical physiological functions^(13)^, the observation that EFAD indicators were adversely associated with growth suggests that FA-based nutritional interventions may be a viable strategy for optimising growth in vulnerable children. Our data affirm similar prior cross-sectional observations among HIV-negative Tanzanian children of 4–6 years old^(18)^ and among thirty-five severely malnourished orphaned children with symptomatic HIV disease from Rumania^(41)^. In this study, EFA sufficiency was positively associated with linear growth in all children in our sample irrespective of perinatal HIV status. In support of our findings and further underscoring the growth disadvantage of CPHIV, LA deficiency has been noted as more prevalent among CPHIV compared to age-matched CHUU^(42)^. Therefore, given the higher morbidity burden due to HIV and malnutrition among CPHIV, they constitute a priority group and the mitigation of EFAD among CPHIV is expected to be especially important to support their growth and potentially cognitive development.

Consistent with observations in the context of metabolic chronic diseases^(4)^, low levels of inflammatory AA and DTA FA were associated with growth advantage in this sample. Further, our data suggest that higher levels of total *n*-6 FA are beneficial for growth in all children. Our finding is consistent with previous cross-sectional reports in this cohort^(20)^, and findings among Tanzanian CHUU^(18)^ but not with findings among Ghanaian children^(17)^. This association, however, was in contrast with the observed inverse relationship of GLA and DGLA (both anti-inflammatory *n*-6 FA) to growth. Of note, GLA is synthesised from LA through the enzymatic action of delta-6-desaturase. The observed inverse association is at odds with the expectation of these FA that are anti-inflammatory at high levels^(43)^. However, the human health effect of GLA is especially complex and the subject of active research. In brief, the relationship of GLA to human health outcomes is varied and inconsistent because of inherent variation in capacity of various human cells/tissues to convert GLA to bioactive metabolites, possible genetic variations in the desaturase and opposing effects of elongation product FA, namely anti-inflammatory DGLA and inflammatory AA^(44,45)^. However, the associations between growth and individual *n*-6 FA, GLA, DGLA, AA and DTA, were mediated by HIV status as magnitude of associations was attenuated and statistically insignificant with adjustment for perinatal HIV status.

### Some n-3 fatty acids influenced growth in HIV-status-dependent manner

Our hypothesis that the relationship of some FA to growth may differ according to dietary requirement for EFA and non-EFA classes within perinatal HIV groups is supported by the relationship of growth to some FA in the SFA, *n*-3 and *n*-9 classes. Overall, these subgroup-specific associations require clarification/confirmation in future studies to determine their clinical/population health relevance.

With respect to *n*-3 FA class, low levels were consistently associated with moderate growth deficit among CHUU. This inverse association was replicated among CHEU was evident, albeit less consistent (i.e. observed for EPA only) but was absent among CPHIV. The importance of *n*-3 sufficiency for growth among CHUU is reinforced by consistent associations for ALA, the sum of EPA and DHA and *n*-3 index. These results directly confirm previously reported observations of positive association between *n*-3 FA and growth among children of <5 years old from Malawi^(46)^. Patterns in our data affirm conclusion of recent systematic review that supplementation efforts to prevent stunting might be most effective by focusing on dietary enrichment for EPA and DHA, especially in children less than 2 years old^(47,48)^. Very few supplementation studies of *n*-3 has been done among children older than 2 years, and meta-analysis of available data did not find significant benefit for growth in older children^(48)^. This study is an important addition to the currently limited evidence base regarding association of *n*-3 levels with attained stature in older children. Of note, although *n*-3 was not associated with growth among CHEU and CPHIV, the fact that higher total PUFA (a composite which includes *n*-3 FA) is associated with higher attained stature in all children suggests that indeed DHA + EPA supplementation could potentially benefit growth for all children in this setting. Data from this study suggest that CHUU who are less medically complex may derive marginally higher benefit from such intervention compared to CHEU and CPHIV^(47,48)^. We found that low oleic acid and low total MUFA predicted better growth over 12 months. The inverse association between oleic acid and growth observed herein confirms previous cross-sectional finding in this sample for CPHIV and CHEU^(20)^. Of note, oleic and nervonic acids have known roles in neurodevelopment, but their relationship to growth has not been clearly established^(49,50)^ and warrants specific elucidation.

The following limitations must be considered in the interpretation of these data. First, FA assessment was done at baseline only and therefore assumes that relative FA profiles remained stable over the study period. Secondly, the initial point of recruitment for many CHUU in this study was a health centre. In this setting, HIV-affected children unlike CHUU are systematically connected to primary care along with its associated benefit for reducing overall morbidity burden, for the purpose of managing HIV disease in the child or their caregiver. Although assessment in all children were made when children were free of apparent symptoms, we cannot exclude the potential that recent illness may have contributed to variations in FA. To the extent that illness-related fluctuation in FA occurred, this fluctuation is expected to be greater among CHUU leading to under-estimation of HIV-status-related differences in growth. Despite these limitations, the prospective longitudinal design of this study with growth measured repeatedly over 12 months allows for delineation of temporal sequences in the relationship of FA to growth. Further strength lies in the inclusion of HIV-affected and HIV-unaffected children which provides a robust comparative framework to elucidate the similarity and differences in FA relationship to growth across HIV groups. Lastly, this study also adds to the body of literature by examining growth and FA in older children.

Further mechanistic studies are needed to properly delineate the health effect of GLA, oleic and nervonic acid on long-term growth of vulnerable children with and without HIV. Here, we confirm a growth disadvantage for CPHIV by school age and adolescent years. This relationship appears driven by the sequela of chronic HIV and was minimally impacted by differences in FA level. However, PUFA was also an independent determinant of growth in this population, and we provide novel data among school-aged children that highlight the opportunity for FA-based nutritional intervention to optimise growth among vulnerable Ugandan children with and without HIV. Specifically, diets low in total SFA high in EFA, low in total *n*-9 and enriched in terms of total PUFA (e.g. fish, chicken, lean meat, peas, beans, oats and nuts) may positively impact growth. Reducing EFAD appears crucial for optimal growth in all children in this setting.

## Supplementary material

For supplementary material accompanying this paper visit https://doi.org/10.1017/S1368980022000611. click here to view supplementary material
